# Mapping the Advanced-Stage Epithelial Ovarian Cancer Landscape Goes Beyond Words: Two Large Language Models, Eight Tasks, One Journey

**DOI:** 10.3390/jcm14072223

**Published:** 2025-03-25

**Authors:** Michela Quaranta, Alexandros Laios, Charlie Rogers, Anastasia Ioanna Mavromatidou, Amudha Thangavelu, Georgios Theophilou, David Nugent, Diederick DeJong, Evangelos Kalampokis

**Affiliations:** 1 Department of Gynaecologic Oncology, ESGO Centre of Excellence for Ovarian Cancer Surgery, St James’s University Hospital, Leeds LS9 7TF, UK; michela.quaranta@nhs.net (M.Q.); um19cr@leeds.ac.uk (C.R.); amudhathangavelu@nhs.net (A.T.); georgios.theophilou@nhs.net (G.T.); david.nugent@nhs.net (D.N.); diederick.dejong@nhs.net (D.D.); 2 Information Systems Lab, Department of Business Administration, University of Macedonia, 54636 Thessaloniki, Greece; bad22021@uom.edu.gr (A.I.M.); ekal@uom.edu.gr (E.K.)

**Keywords:** epithelial ovarian cancer, natural language processing, transfer learning, operative notes, RoBERTa, GatorTron

## Abstract

**Background/Objectives:** The advancement of natural language processing (NLP) technologies has transformed various sectors. However, their application in the healthcare domain, particularly for analysing clinical notes, remains underdeveloped. We investigated the use of deep neural networks, specifically transformer-based models, to predict intraoperative and post-operative outcomes related to advanced-stage epithelial ovarian cancer cytoreduction (aEOC) using unstructured surgical notes. **Methods:** We evaluated the performance of RoBERTa, a general-purpose language model, and GatorTron, a domain-specific model, across eight binary classification tasks using the same dataset. The dataset consisted of 560 surgical records from patients with aEOC who underwent cytoreductive surgery at a tertiary UK reference centre. Predictive outcomes were converted into binary features to facilitate classification tasks. To enhance the contextual information available to the models, textual data from “operative findings” and “operative notes” were concatenated. **Results:** Our findings highlight the tangible benefits of employing domain-specific language models for clinical text analysis. GatorTron generally outperformed RoBERTa across most predictive tasks, underscoring the advantages of domain-specific pretraining for understanding medical terminology and context. Both models struggled to predict certain outcomes, particularly those involving post-operative events like major complications and length of hospital stay, despite adjustments in hyperparameters and training strategies. This limitation suggests that operative text alone may not sufficiently capture the complexities of post-operative recovery. **Conclusions:** These findings have valuable implications for developing medical AI systems to improve the delivery of modern aEOC healthcare.

## 1. Introduction

Advanced stage epithelial ovarian cancer (aEOC) remains clinically challenging as a leading cause of gynecological cancer mortality [1]. Patients often present with non-specific symptoms, and the disease is frequently diagnosed at a late stage. Optimal surgical cytoreduction combined with platinum-based chemotherapy is the cornerstone of therapy, with the extent of tumour debulking being a strong predictor of overall survival [2]. However, major cytoreductive procedures carry considerable morbidity, making risk stratification and perioperative management paramount for patient counselling and improved outcomes [3].

The COVID-19 pandemic was a catalyst for a rapid digital transformation in healthcare, reflected in the widespread adoption of electronic health records (EHRs) [4]. Despite the wealth of clinical information in EHRs, up to 80% of these data stored in unstructured text, such as clinical notes, operative findings, and discharge summaries, are not used [5]. Although traditional statistical approaches often overlook these unstructured data, leveraging natural language processing (NLP) could offer additional insights from free-text clinical documents [6].

Language models have been applied in diverse healthcare contexts. The deployment of large language models (LLMs) like GPT-3 and GPT-4 in the medical field has opened new avenues for patient care and medical research. These models assist in tasks such as automated medical record summarisation and clinical decision support, demonstrating the transformative potential of LLMs in healthcare [7]. Their performance varies depending on factors such as domain-specific training data and the complexity of clinical tasks. GatorTron, a domain-specific language model designed for clinical text, has shown promise in several healthcare-related tasks [8]. RoBERTa, built on the Bidirectional Encoder Representations from Transformers (BERT) architecture, has also demonstrated strong performance in general text classification, including biomedical applications [9]. We previously demonstrated that RoBERTa-based classifiers, when employed to process unstructured operative notes, outperformed traditional risk factor-based models in the prediction of surgical outcomes [10].

However, few direct comparisons between these two models exist [11], and not within gynaecological oncology and, more specifically, in the context of advanced EOC cytoreductive surgery. This gap could be bridged by combining the NLP work with domain-specific knowledge and deep insight into clinical workflows. To address this, we conducted a head-to-head comparison of GatorTron and RoBERTa for eight binary classification tasks associated with perioperative outcomes and post-operative morbidity in aEOC. By integrating text from operative notes with additional operative findings, we hypothesised that our selected NLP-based neural network models could identify signals predictive of key risk factors, including the likelihood of complete cytoreduction, the length of hospital stay, the estimated blood loss, the operative time, the need for intensive treatment unit admission, and post-operative complications. Ultimately, our goal is to demonstrate the potential of modern NLP techniques in improving perioperative risk management and standardising the reporting of key surgical outcomes after advanced cytoreduction of EOC.

## 2. Materials and Methods

### 2.1. Study Design and Population

We reviewed the hospital EHRs to identify women with aEOC (FIGO stage III or IV) who underwent cytoreductive surgery at a tertiary UK referral centre between January 2014 and December 2019. The EHR dataset consisted of 560 surgical notes detailing surgical findings. An aEOC clinical database, developed internally, was integrated with the EHR system to provide access to both discrete and engineered data [12]. Institutional research ethics board approval was granted by the Leeds Teaching Hospitals Trust (23/NE/0229/328779/12.01.24), and informed written consent was obtained from all participants. This study was registered with the UMIN Clinical Trials Registry (UMIN000049480). Exclusion criteria included missing operative notes, lack of histopathological aEOC confirmation, and incomplete post-operative follow-up data.

### 2.2. Data Extraction and Processing

A customised pipeline was developed to extract and pre-process data from the institutional EHR system. Structured fields—such as patient demographics, date of surgery, date of discharge, and standard laboratory values—were collected using query-based methods. The dataset originally contained 113 fields; however, this was reduced to 10 fields to focus on the unstructured text and most relevant data. These included two text fields (used as model inputs) and eight predictive outcomes (used as output variables) (Table 1). To address the variability in documentation styles amongst different surgeons, we applied synonym standardisation for text normalisations and observed some mild heterogeneity in terminology usage.

The unstructured text primarily consisted of the following:

**Operative Notes**: Detailed narratives documented by the surgeon describing the operative approach, location and extent of disease, specific manoeuvres undertaken at cytoreduction, and immediate surgical events and/or instructions.

**Operative Findings**: Additional text-based reports outlining tumour distribution and the involvement of various anatomical structures.

These narratives were concatenated into a unified corpus for each patient to allow for NLP analysis and improve model performance. All clinical features were transformed into binary to ensure a uniform comparison. For continuous variables (e.g., length of stay, procedure time, blood loss, and time between surgery and treatment), a median split was applied. Binary variables included whether the patient had a complete cytoreduction, major post-operative complications, ITU admission, and CA125 levels post-treatment. To address imbalanced fields, class weights were adjusted during the model training using this formula:

$$\mathrm{Weight}=\frac{n_{\mathrm{samples}}}{\left(n_{\mathrm{classes}}\cdot \mathrm{np}.\mathrm{bincount}\left(y\right)\right)}$$

The surgical outcomes included eight binary classification tasks reflecting peri-operative and post-operative parameters:

1. Complete Cytoreduction: Whether the surgeon recorded no visible residual disease at the end of the procedure.
2. Length of Stay: Whether the post-operative stay extended beyond 7 days (median), a clinically meaningful threshold to capture extended hospitalisation.
3. Operative Time: Documented in minutes.
4. Estimated Blood Loss: Documented in millilitres.
5. Intensive Care Unit (ICU) Admission: Whether the patient required ICU admission in the immediate post-operative period.
6. Clavien-Dindo Grade 3–5 post-operative complications: Complications that necessitated surgical, endoscopic, or radiological intervention (grade 3), that necessitate critical organ support (grade 4), or that resulted in death (grade 5) [13].
7. Time between surgery and end of treatment: Indicating potential treatment delays.
8. End-of-Treatment CA125: Whether the patient’s serum CA125, measured upon completion of chemotherapy or follow-up, remained elevated or not (≥35 U/mL).

Structured and unstructured entries in operative discharge summaries and follow-up notes were thoroughly reviewed by two clinicians (AL, DDJ) to establish ground truth labels. Identifiers and protected health information were removed to maintain patient confidentiality in line with institutional guidelines.

### 2.3. Large Language Model Architectures

GatorTron is a domain-specific Transformer-based language model developed from clinical notes, PubMed articles, and Wikipedia. It is designed to capture nuances of medical terminology [8].

RoBERTa (Robustly Optimised BERT Pretraining Approach) was chosen for its robust optimisation and dynamic masking technique, which enhances generalisation and reduces dependency on word order. It is trained on large datasets and has demonstrated superior performance across various NLP benchmarks [14]. RoBERTa has also been applied to detect negations in Dutch clinical texts, showing improved accuracy of clinical information extraction [15]. This suggests that RoBERTa, although not specifically designed for healthcare, can be fine-tuned for medical tasks, whilst specialised models like GatorTron retain their enhanced accuracy and efficiency in medical settings.

RoBERTa was trained and fine-tuned using the Simple Transformers library. Due to its computational cost, GatorTron was trained for 40 epochs using the default Simple Transformers learning rate ($4.00\cdot 10^{-5}$). The same loss function (cross-entropy loss) was used for consistency. Both models were trained on an 80–20 train–test split. Class weights for imbalanced variables were incorporated during training to mitigate bias. Key hyperparameters are presented in Table 2.

### 2.4. Performance Metrics and Statistical Analysis

We assessed each model’s discriminative ability using the area under the receiver operating characteristic curve (AUROC). In addition, we computed accuracy, precision, recall, F1-score, Matthew’s correlation coefficient (MCC), and the area under the precision-recall curve (AUPRC). We evaluated differences in performance using DeLong’s test for AUROC comparisons where appropriate. Statistical significance was set at p<0.05. Each metric was calculated for every binary classification task, allowing for a thorough comparison of the models’ strengths and weaknesses.

## 3. Results

**Figure 1:**
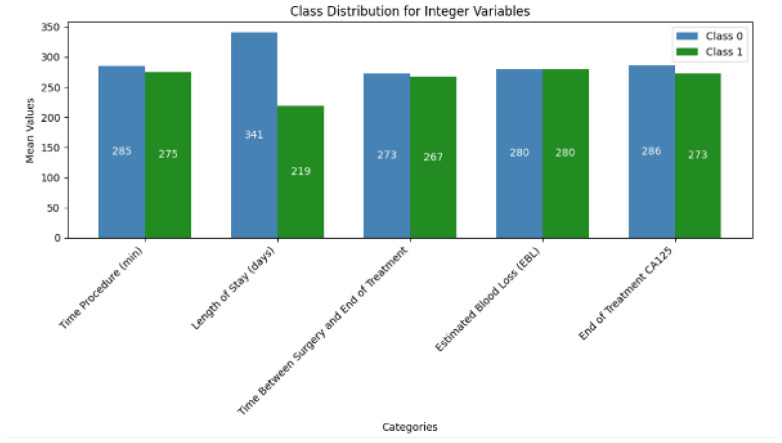
Class distribution of Integer features.

The descriptive statistics of the continuous variables are summarized in Table 3. The integer to binary transformation was performed by using the median value as the threshold. This concluded in relatively balanced distribution between most variables (Figure 1).

The concatenated field that was created from the merge of operative findings and operative notes is referred to as “operative text”. The descriptive statistic of the word counts for the three text fields are shown on Table 4.

**Figure 2:**
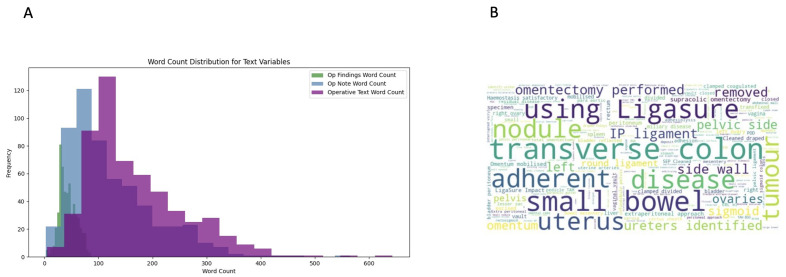
(**A**) Word count distribution of text variables. The variation reflects the different purposes of the fields. (**B**) Word cloud visualisation of the concatenated texts extracted from operative notes and operative findings. More frequent terms appear larger.

The word count distribution showed significant variation between operative findings and operative notes fields, with the operative findings typically having shorter word counts (30–35 words). In contrast, operative notes had a wider spread, peaking around 100–150 words and extending up to 565 words in length (Figure 2A). To gain insights into commonly used terms, word clouds were generated (Figure 2B).

During model convergence, GatorTron displayed a steady increase in validation AUROC with fewer epochs, whilst RoBERTa required more tuning cycles. For RoBERTa, using only the text field of “operative findings”, performance accuracies for most outcomes were slightly above 50%, which is a little better than random chance. Similar observations were made for GatorTron. Therefore, the “operative text” was used as the data input. All variables except ICU admission and major post-operative complications were predicted by a 40-epoch training with the default learning rate of $4.00\times 10^{-5}$. The two imbalanced fields underwent weight readjustment to improve model performance. Table 5 and Table 6 summarise the performance metrics for RoBERTa and GatorTron across the eight binary tasks, respectively.

GatorTron consistently outperformed RoBERTa, with the most pronounced differences observed in classification tasks requiring greater domain-specific knowledge, such as predicting the need for ICU admission, identifying post-operative CD grade 3–5 complications, and detecting the possibility of incomplete cytoreduction. Across all tasks, GatorTron’s AUROC values showed statistically significant improvements compared to RoBERTa (p<0.05).

**Figure 3:**
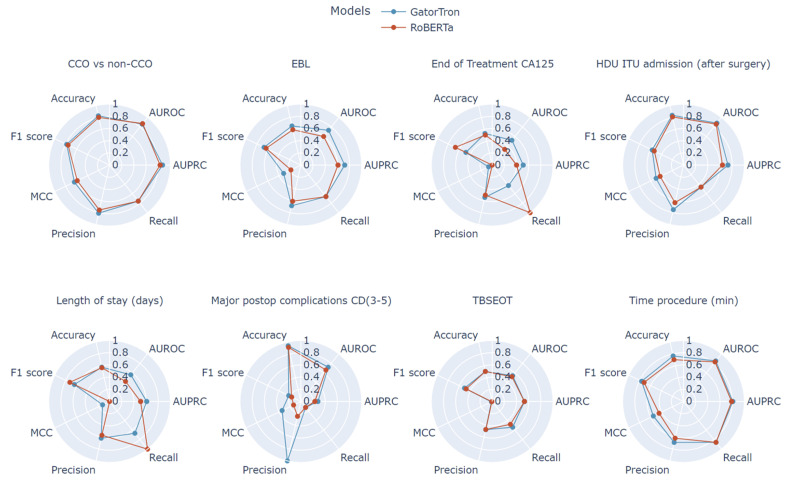
Radar plots comparing performance between RoBerta and GatorTron models for all examined clinical tasks using Matthew’s correlation coefficient (MCC), recall, precision, F1 score, accuracy, area under precision–recall curve (AURPC), and area under receiver operating characteristic curve (AUROC).

The greatest improvement was for the ICU admission outcome (AUROC +0.06), which relied heavily on textual indicators such as extensive organ resection or blood transfusion, both of which were better captured by GatorTron’s domain-specific embeddings (Figure 3).

In addition to quantitative performance, the unstructured concatenated text revealed several unexpected correlations when analysed qualitatively. Shorter operative times did not always translate to minimal disease burdens as some fast-track surgeries involved critically unstable patients or limited attempts at cytoreduction owing to borderline feasibility. Similarly, while one might anticipate that higher EBL would correlate with prolonged hospital stay, certain cases displayed speedy recovery despite substantial intraoperative bleeding. These nuances underline the value of systematic text analysis, which can unveil latent factors influencing EOC surgical outcomes.

## 4. Discussion

This work showcases the growing intersection of clinical medicine and AI-driven NLP that holds vast promise for improving surgical oncology. Herein, we present the first comparison between RoBERTa and GatorTron in the context of aEOC. Our results demonstrate that domain-specific NLP architectures like GatorTron possess superior discriminatory power for predicting multiple perioperative and post-operative outcomes in cytoreductive surgery. The novel integration of operative notes with operative findings into a single textual input enabled enhancing the model performance. By employing unstructured free-text fields that are typically underutilised or entirely overlooked, GatorTron outperformed the more general-purpose RoBERTa model in nearly every classification task examined. For instance, in predicting procedure time, GatorTron achieved an accuracy of 76.6% and an MCC of 0.550, whereas RoBERTa attained 70.3% accuracy and 0.446 MCC.

There are several potential explanations for GatorTron’s superior performance. First, its training on large volumes of clinical text exposes it to unique context not seen in generic language corpora. Consequently, GatorTron more readily identifies conceptually relevant phrases such as “optimal cytoreduction”, “residual nodules”, or “significant ascites”. Secondly, the model’s domain-specific embedding space allows for the improved handling of ambiguous terms (e.g., “debulking” or “interval cytoreduction”), which may be interpreted differently by general-purpose models lacking clinical context. Finally, aEOC operative reports often contain complex, highly technical descriptions of procedures or tumour spread. GatorTron’s capacity to incorporate domain-sensitive relationships likely offers fine-grained predictions. That said, to improve model performance, more precise definitions of surgical subprocedures—such as the various types of peritonectomy—are required [16], or the use of standardised operative templates is warranted [17]. RoBERTa struggled more with domain-specific terms leading to lower performance in tasks requiring a deeper clinical understanding. These model performance variations may potentially benefit from multimodal data integration, such as laboratory values and imaging findings.

Beyond comparing these two models, our work underscores the importance of structured and standardised reporting of perioperative details. The combined textual data from operative notes and findings proved more effective for predictive modelling than either alone, suggesting that future informatics in aEOC (and in other oncological fields) should facilitate the integration of multiple textual sources. By capturing potential confounders such as tumour location, infiltration depth, and surgical complexity, text-based approaches can offer a holistic representation of the intraoperative scenario than simple numeric fields like duration of surgery or estimated blood loss.

Despite these promising results, both models encountered challenges in predicting certain outcomes, particularly those involving post-operative events like major complications and length of hospital stay. This limitation suggests that capturing the complexities of post-operative recovery solely from operative text may not be sufficient. Such picked counterintuitive correlations in the surgical outcomes could inform future research and clinical decision-making.

In this work we prioritised predictive performance over model interpretability. Nevertheless, it remains crucial for clinicians to trust the predictions AI systems make. Techniques like SHAP (SHapley Additive exPlanations) or LIME (Local Interpretable Model-Agnostic Explanations) could be used to highlight words or phrases in the surgical notes that contribute most to predictions. Model explainability was embodied in our previous work [10]. As NLP tools transition from research to real-world application, inherent biases to in-training data cannot be neglected. We propose a hybrid approach where AI-driven predictions are reviewed alongside clinician assessments to prevent automated decision-making biases.

### 4.1. Clinical Implications

By combining both quantitative and qualitative data, our work offers guidance towards the integration of NLP systems that provide clinicians with evidence-based, risk-adjusted patient counselling [18,19]. Integrative qualitative analysis revealed unexpected nuances, which underline the value of systematic text interrogation to unveil latent factors influencing aEOC surgical outcomes. From a practical viewpoint, the capacity to accurately stratifying perioperative risks in aEOC has clear implications for patient counselling, resource allocation, and post-operative care planning. For instance, an NLP system integrated into the electronic health environment could flag high-risk cases for additional monitoring allowing surgeons to intervene earlier or perhaps guide surgeons in discussing potential complications and recovery trajectories with patients [20]. This approach, embedded into an interactive Clinical Decision Support System might drive more personalised surgical plans, optimising the likelihood of complete cytoreduction whilst mitigating undue harm. Engagement of surgical teams via workshops to familiarise them with AI-generated insights can ensure informed adoption. These steps align with broader efforts to operationalise AI in surgical oncology.

Our study highlights prospects for future AI applications in aEOC more broadly. One intriguing application is the development of ovarian cancer-specific chatbots that could field patient questions on perioperative events, expected complications, or post-operative recovery. By drawing on real-world operative data and outcomes, such a chatbot could deliver targeted, evidence-based advice, aiding both clinicians and patients throughout the treatment continuum.

### 4.2. Strengths and Novel Contributions

The main strength of the study is the head-to-head comparison of state-of-art GatorTron and RoBERTa models for aEOC-related perioperative predictions, contributing to the ongoing discourse around domain-specific versus general-purpose language models in healthcare, based on same input data. We explored not only immediate measures of surgical success but also post-operative morbidity, resource utilisation (ICU admission), and biochemical endpoints (end-of-treatment CA125), thereby painting the bigger picture of the perioperative aEOC journey. By using actual operative notes and findings from a high-volume tertiary referral centre, our results are highly relevant to clinical practice, with potential for direct translational impact. Counterintuitive associations highlighted the complexity of surgical outcomes and illustrated how NLP can uncover nuanced insights that might not conform to standard assumptions. Our qualitative analysis indicated how, for example, shorter operative times were associated with unstable patients rather than straightforward procedures adding to recently published evidence [21]. It will be interesting to propose a future study where domain experts can perform annotation tasks for outlier cases to further understand underlying factors.

### 4.3. Limitations

Several limitations warrant consideration. First, a single-institution study with a modest sample size may limit the generalisability of findings. Nevertheless, replication in diverse settings is essential to affirm the broader utility of these models. Second, the manual labelling performed by two clinicians was resource-intensive and might have introduced subjective bias. Future research could explore semi-automated labelling or active learning to reduce reviewer burden [22]. Third, both models could be vulnerable to domain shifts, i.e., unexpected changes in documentation style or use of different “macro” templates, potentially affecting model accuracy. There was an exclusive reliance on the operative notes as input data. These narratives might not capture all factors affecting post-operative complications or time between surgery and end of treatment. An end-to-end risk prediction tool should better combine both numeric and free-text information for maximal predictive precision. We recognise the relatively small dataset albeit both domain-specific LLMs are pre-trained and they do not necessarily require a large dataset for modelling. We considered data augmentation strategies such as back translation, but they were not implemented due to the complexity of medical terminology.

### 4.4. Future Directions

Based on our findings, we propose the prospective implementation of GatorTron or similar domain-specific models into real-time clinical decision support tools within EHR platforms to help surgeons identify patients at higher risk of morbidity or incomplete cytoreduction. Although we prioritised predictive performance over interpretability, the adoption of model explainability might assist clinicians in understanding the basis for aEOC-specific predictions [23]. This research is expected to evolve including external validation, multimodal NLP models, and alternative explainability techniques.

For effective clinical adoption, integration into Hospital EHRs is essential. Model deployment as a real-time background process could continuously analyse new surgical notes for predictive insights. These considerations may be challenged by computing costs or institutional resistance through pilot programs.

Finally, this type of work can potentially pave the way towards the development of a bespoke, proprietary, domain-specific chatbot capable of synthesising textual EHR data. Such a system could join similar efforts [24,25] and act as a first point of contact for patient questions, bridging the gap between appointments and streamlining communications with clinical teams.

## 5. Conclusions

Our study confirms that domain-specific NLP models, exemplified by GatorTron, can effectively employ unstructured EHR data to predict a broad range of important clinical outcomes at aEOC cytoreductive surgery. In comparison, a general-purpose model such as RoBERTa displayed inferior performance, highlighting the value of clinical context and domain-specific language embeddings. Investing in domain-specific architectures, robust data extraction protocols, and comprehensive outcome metrics can yield actionable intelligence that informs the delivery of high-quality, patient-centric care in EOC, and more. Our work lays the groundwork for developing advanced AI applications, including chatbots.

## Figures and Tables

**Table 1:** Selected dataset fields.

Text Fields	Patient Characteristics	Original Data Type
Operative Notes (Op Note)	Time procedure (minutes)	Binary
Operative Findings (Op Findings)	Length of stay (days)	Binary
	Time between surgery and end of treatment	Binary
	Intensive Care Admission (after surgery)	Integer
	Estimated Blood Loss (EBL)	Integer
	End of Treatment CA125	Integer
	Complete cytoreduction vs non-complete cytoreduction	Integer
	Major post-operative complications Clavien Dindo (CD) (3–5)	Integer

**Table 2:** Hyperparameter settings for RoBERTa and GatorTron fine-tuning.

	RoBERTa	GatorTron
Epochs	40, 60	40
Learning Rate	$4.00\cdot 10^{-5}$ (default), $1.00\cdot 10^{-7}$, $1.00\cdot 10^{-5}$	$4.00\cdot 10^{-5}$ (default)
Loss Function	Cross-entropy loss	Cross-entropy loss

**Table 3:** Descriptive statistics for key surgical and clinical parameters.

	Time Procedure (Minutes)	Length of Stay (Days)	Time Between Surgery and End of Treatment	Estimated Blood Loss (EBL)	End of Treatment CA125
Count	560	560	540	560	559
Mean	170.39	8.32	91.25	524.50	61.57
Median	150	7	73	425	13
Std	77.55	8.65	85.14	387.78	347.40
Min	30	2	0	50	2
25%	115	6	58	300	8
75%	205	9	119.5	600	23
Max	600	174	1325	4500	5646

**Table 4:** Descriptive statistics for operative text, operative findings, and operative notes.

	Operative Text	Operative Findings	Operative Note
Count	560	560	560
Mean	165.12	43.12	122.98
Median	139.5	41	99
Std	90.84	16.44	80.44
Min	0	0	0
25%	101	31.75	64
75%	214	54.25	165
Max	643	86	565

**Table 5:** RoBERTa evaluation metrics—operative text with 40-epoch training.

Target Field	Accuracy	Recall	Precision	F1-Score	AUPRC	AUROC	MCC
CCO vs. non-CCO	0.802	0.756	0.756	0.756	0.83	0.87	0.589
EBL	0.595	0.661	0.609	0.634	0.61	0.60	0.182
End of treatment CA125	0.505	1.000	0.505	0.671	0.40	0.33	0.000
Length of stay	0.568	1.000	0.568	0.724	0.51	0.42	0.000
Time between surgery and end of treatment	0.505	0.480	0.471	0.475	0.53	0.52	0.006
Time procedure	0.703	0.857	0.618	0.718	0.79	0.83	0.446
HDU/ITU admission	0.811	0.462	0.632	0.533	0.64	0.86	0.426
Major postop complications	0.910	0.125	0.250	0.167	0.23	0.66	0.133

**Table 6:** GatorTron evaluation metrics—operative text with 40-epoch training.

Target Field	Accuracy	Recall	Precision	F1-Score	AUPRC	AUROC	MCC
Time procedure	0.766	0.857	0.689	0.764	0.81	0.85	0.550
Length of stay	0.577	0.667	0.618	0.641	0.61	0.56	0.127
Time between surgery and end of treatment	0.505	0.540	0.474	0.505	0.53	0.54	0.014
HDU/ITU admission	0.838	0.462	0.750	0.571	0.73	0.88	0.500
EBL	0.658	0.661	0.684	0.672	0.72	0.73	0.314
End of treatment CA125	0.532	0.429	0.545	0.480	0.51	0.52	0.066
CCO vs. non-CCO	0.829	0.756	0.810	0.782	0.87	0.86	0.642
Major postop complications CD (3–5)	0.937	0.125	1.000	0.222	0.28	0.72	0.342

## Data Availability

Data presented in this study are available on request from the corresponding author.
